# Prognosis and Complications of Diabetic Patients Undergoing Isolated Coronary Artery Bypass Surgery

**DOI:** 10.5935/1678-9741.20160002

**Published:** 2016

**Authors:** Karen Alcantara Queiroz Santos, Bharbara Berto, Alexandre Gonçalves Sousa, Fernando Augusto Alves da Costa

**Affiliations:** 1 Universidade Anhembi Morumbi, São Paulo, SP, Brazil.; 2 Hospital Beneficência Portuguesa de São Paulo, São Paulo, SP, Brazil.

**Keywords:** Diabetes Mellitus, Coronary Artery Bypass, Diabetes Complications, Cardiac Surgical Procedures, Comparative Study

## Abstract

**Objective:**

Compare the prognosis and complications of diabetic and non-diabetic patients undergoing isolated coronary artery bypass surgery at a hospital with a high surgical volume.

**Methods:**

Data of patients who underwent coronary artery bypass surgery from June 2009 to July 2010 were analyzed. We selected diabetic and non-diabetic patients and evaluated their postoperative and long-term prognosis based on clinical complications. To reduce the disparity within the sample, statistical analyses were performed using propensity scores.

**Results:**

We included 2,688 patients who underwent coronary artery bypass surgery; 36% of them had diabetes, their mean age was 62.1±9.49 years and 70% (1,884) of them were men. Patients with diabetes were older (63±9 years *vs.* 61±10 years; *P*<0.001), more often obese (BMI>25 kg/m^2^: 70.7% *vs.* 64.5%; *P*<0.001), dyslipidemic (50.4% *vs.* 41.1%; *P*<0.001), hypertensive (89.2% *vs.* 78.7%; *P*<0.001), and presented chronic renal failure (8.3% *vs.* 3.8%; *P*<0.001). They also presented higher rates of acute renal failure (5.6% *vs.* 2.7%, *P*<0.001), infection (11.4% *vs.* 7.2%, *P*<0.001) and mortality after one year (9.1% *vs.* 5.6%, *P*<0.001). Pneumonia was more common among patients with diabetes (7.7% *vs.* 4.0%, *P*<0.001). According to propensity scoring, 430 patients (215 diabetics and 215 non-diabetics) had a mean age of 61.3±8.97 years, and 21.2% (91 of 430) were women. However, diabetes was not an independent factor for poor prognosis.

**Conclusion:**

Patients with diabetes were at higher risk for postoperative complications and mortality after undergoing coronary artery bypass surgery. However, diabetes did not explain the poor prognosis of these patients after pairing this factor with the propensity score.

**Table:** 

**Abbreviations, acronyms & symbols**	
AMI	= Acute myocardial infarction
BMI	= Body mass index
CABG	= Coronary artery bypass grafting
CHF	= Congestive heart failure
CKD	= Chronic kidney disease
CVD	= Cardiovascular disease
DM	= Diabetes mellitus
HTN	= Hypertension
IOT-Re	= Reintubation rates

## INTRODUCTION

Diabetes mellitus (DM) is considered to be one of the most prevalent diseases in the western world, constituting a true epidemic in urbanized societies. Its importance is related not only to the increasing incidence of the disease, but also to its extremely close relationship with atherosclerotic disease, which has a great impact on morbidity and mortality worldwide. Estimates of the World Health Organization show that worldwide prevalence of this disease may have an increase of 114% during the next 20 years, leading to the emergence of 330 million new cases^[1]^. Brazil is following this global trend, and is included among the 10 countries with the highest absolute number of individuals with DM^[2]^. In this context, it is necessary to remember that one of the main causes of mortality related to DM is cardiovascular disease (CVD), especially coronary artery disease, accounting for 60% to 80% of deaths among adults.

Patients diagnosed with DM represent approximately 25% of all patients undergoing surgical or percutaneous revascularization procedures. They have a worse prognosis in relation to coronary heart disease compared with patients who do not have DM, as well as different prognoses when treated by percutaneous intervention with catheters or surgery. Coronary artery bypass grafting (CABG) is nowadays the preferable revascularization treatment of diabetic patients^[3,4]^.

In relation to surgical treatment, several studies revealed higher morbidity and perioperative mortality rates among patients with DM undergoing CABG. This finding is probably related to the occurrence of perioperative myocardial infarction, infections, respiratory failure, renal and cerebral complications, all of which prolong hospitalization in these patients^[5-7]^. Moreover, the presence of DM is considered to be an independent risk factor for postoperative mortality after CABG, with an odds ratio of 1.73 for cardiovascular death and 2.94 for overall mortality^[8,9]^. In Brazil, a study shows mortality as high as 11.8% in diabetic patients^[10]^. But another one with octogenarian patients did not found differences in mortality^[11]^.

This study aims to compare the prognosis and complications of diabetic patients with non-diabetic undergoing isolated CABG at a hospital with a high surgical volume.

## METHODS

Patient evaluation was performed by collecting prospective data on patients undergoing CABG at our hospital from June 2009 to July 2010. Three senior nurses at the hospital were trained to collect these data using prespecified settings. Patients were included in the database if they fulfilled the following inclusion criteria: they were consecutive patients aged 18 years or older (no restrictions on sex or race) and had undergone CABG. Patients were excluded if they had undergone any other surgery, including cardiac surgery, in which isolated CABG or congenital cardiac surgery was not performed.

From this sample, patients who underwent isolated CABG were selected, and this population was then divided into those with DM and those without DM. For these groups, postoperative prognoses with clinical complications, as well as long-term outcomes, were evaluated by telephone interviews; these interviews were conducted from the 30^th^ day after surgery to one year after surgery.

Among the clinical complications that occurred following CABG, the following variables were analyzed: perioperative acute myocardial infarction (AMI), neurological complications, pulmonary complications, infectious complications, cardiac arrhythmias, and multiple organ failure occurring within 30 days after the surgery.

### Clinical complications were defined as follows:

- Perioperative AMI was defined as: prolonged pain (> 20 minutes); typical pain not improved by nitrates; elevation of cardiac enzymes (CK-MB or troponin > 0.2 micrograms/ml); or a new alteration in cardiac mobility or electrocardiogram series (at least two) showing new changes in the ST/T segment or new Q waves of at least 0.03 seconds or more than one-third of QRS in at least two contiguous leads;
- Stroke (cerebrovascular accident) was defined as the persistence of motor deficit for more than 72 hours or coma for more than 24 hours;
- Acute renal failure was defined as serum creatinine level greater than 2.0 mg/dl, two times greater than the preoperative level, or the necessity of dialysis by any method;
- Infectious processes were defined as: Mediastinitis (defined as deep infection involving muscles, bone and/or mediastinum, fulfilling the following conditions: 1. open wound with tissue excision, 2. positive cultures and 3. treatment with antibiotics); surface of thoracotomy (infection involving thoracotomy and parasternal region fulfilling the following conditions: 1. open wound with tissue excision, 2. positive cultures and 3. treatment with antibiotics); superficial leg incision (infection involving the site of dissection of mediastinal veins fulfilling the following conditions: 1. open wound with tissue excision, 2. positive cultures and 3. treatment with antibiotics); and infections related to venous catheters and the urinary tract.
- Pulmonary complications were defined as prolonged mechanical ventilation (i.e., the need for ventilation for more than 48 hours in the postoperative period), acute respiratory distress syndrome, pulmonary embolism or pneumonia (diagnosed by the following criteria: positive cultures of sputum, blood or pleural fluid; empyema; or chest X-ray with new infiltrates);
- Cardiac arrhythmias that required intervention comprised atrial fibrillation, atrial flutter, paroxysmal supraventricular tachycardia and bradyarrhythmias;
- Failure of multiple organs was defined as two or more systems with compromised functioning.

All the hospital's cardiac surgery teams agreed to provide data for the development of the database. The logistic EuroSCORE was individually calculated for all patients (preoperative risk score) and expressed as the average of the groups.

The project was submitted and approved by the Ethics Committee of the Institution with CAEE number 43817015.2.0000.5483. Because this was a retrospective database survey, an informed consent form was not given to the patients. The data obtained from the database were confidentially treated by the research center.

### Statistical considerations

Initially, all variables were descriptively analyzed. For quantitative variables, analyses were carried out by observing minimum and maximum values and calculating the mean and standard deviation. For qualitative variables, absolute and relative frequencies were calculated.

To compare the averages of the two groups, we used Student's t test^[12]^, and when the assumption of data normality was rejected, we used the nonparametric Mann-Whitney *U* test^[12]^. To test the homogeneity between proportions, we used the chi-square test or the Fisher's exact test^[12]^.

### Propensity Scoring

To minimize the bias resulting from collecting data at different times and in a nonrandomized way, and to balance the characteristics of patients within the sample, patients in the DM groups were combined using a propensity score, defined as the conditional probability of belonging to a determined group. This procedure corresponds to selecting patients from the diabetic group who were similar to patients from the non-diabetic group.

First, a logistic regression model^[13]^ was created using the variable group as the dependent variable. The most relevant confounders [sex, body mass index (BMI in kg/m^2^), chronic kidney disease (CKD), hypertension (HTN), previous cerebrovascular accident, congestive heart failure (CHF), endotracheal reintubation, and cardiopulmonary bypass support] were used as predictive factors, and the corresponding tolerance margin was a 0.05 logit. Next, paired cases were selected based on the propensity logistic regression score. This model was built based on a sample of patients paired by a propensity of 01:01, without substitution or repetition. The significance level used for the tests was 5%.

## RESULTS

The study included 2,688 diabetic and non-diabetic patients who underwent CABG, with an average age of 62.1 years and 70% (1,884) males (Table 1). Patients with DM accounted for 36% (990 of 2,688) of the total population. Patients with DM were older on average (63±9 years *vs.* 61±10 years for non-diabetics; *P*<0.001), more were men (64.6% diabetics *vs.* 73.3% non-diabetics; *P*<0.001), and more were overweight, with the majority having a BMI>25 kg/m^2^ (70.7% diabetics *vs.* 64.5% non-diabetics, *P*<0.001). In addition, patients with DM were more often former smokers (42.0% *vs.* 38.4% of non-diabetics; *P*<0.001), dyslipidemic (50.4% *vs.* 41.1% of non-diabetics; *P*<0.001) and presented CKD (8.3% *vs.* 3.8% non-diabetics, *P*<0.001). Of these, the majority of patients were being treated with dialysis (28.1% of patients with DM *vs.* 14.1% of non-diabetics; *P*<0.043), had HTN (89.2% of patients with DM *vs.* 78.7% of non-diabetics; *P*<0.001), more often had a history of cerebrovascular accident (7.4% of patients with DM *vs.* 4.0% of non-diabetics; *P*=0.001), peripheral arterial disease (6.0% of patients with DM *vs.* 4.0% non-diabetics; *P*=0.017) and were more likely to present CHF (3.3% of patients with DM *vs.* 1.2% non-diabetics; *P*<0.001). The expected mortality rate calculated by EuroSCORE was also higher in patients with DM: 2.6±2.4 compared with 2.2±2.0 in non-diabetics (*P*<0.001).

**Table 1:** Descriptive values [average ± standard deviation or n (%)] of preoperative variables according to the study group.

	Original Cohort (n=2,688)		
	Diabetes		*P* value
Variables	No (n=1,698)	Yes (n=990)	
Age	61±10	63±9	<0.001^[1]^
Male sex	1,245 (73.3)	639 (64.6)	<0.001^[2]^
Body mass index (kg/m^2^)			
< 25	584 (35.5)	283 (29.3)	<0.001^[2]^
25-30	774 (47.1)	427 (44.3)	
>30	285 (17.4)	255 (26.4)	
Smoking history:			
Smokers	318 (18.7)	104 (10.5)	<0.001^[2]^
Former smokers	652 (38.4)	416 (42.0)	<0.001^[2]^
Never smoked	728 (42.9)	470 (47.5)	<0.001^[2]^
Dyslipidemia	697 (41.1)	499 (50.4)	<0.001^[2]^
Chronic kidney disease	64 (3.8)	82 (8.3)	<0.001^[2]^
In dialysis (n=146)	9 (14.1)	23 (28.1)	0.043^[2]^
Hypertension	1,336 (78.7)	883 (89.2)	<0.001^[2]^
Previous cerebrovascular accident	67 (4.0)	73 (7.4)	0.001^[2]^
Chronic obstructive pulmonary disease	128 (7.5)	56 (5.7)	0.062^[2]^
Peripheral arterial disease	67 (4.0)	59 (6.0)	0.017^[2]^
Cerebrovascular disease	27 (1.6)	21 (2.1)	0.316^[2]^
Serum creatinine	1.3±0.6	1.4±0.9	<0.001^[1]^
Prior CABG	20 (1.2)	18 (1.8)	0.175^[2]^
Prior acute myocardial infarction	801 (47.2)	442 (44.7)	0.205^[2]^
Congestive heart failure	21 (1.2)	33 (3.3)	<0.001^[2]^
Arrhythmia	81 (4.8)	54 (5.5)	0.433^[2]^
Ejection fraction	65.6±11.9	63.5±12.4	0.005^[1]^
EuroSCORE logistics	2.2±2.0	2.6±2.4	<0.001^[3]^

[1] Descriptive level of probability determined by the Student's t-test.

[2] Descriptive level of probability determined by the chi-square test.

[3] Descriptive level of probability determined by the nonparametric Mann-Whitney *U* test.

In relation to medical treatment, diabetics had the highest rates of previous use of medications such as calcium-channel blockers (22.8% of patients with DM *vs.* 18.1% of non-diabetic patients; *P*=0.003), angiotensin-converting enzyme inhibitors (50.5% of patients with DM *vs.* 46.5% of non-diabetic patients; *P*=0.046) and angiotensin II receptor blockers (20.2% of patients with DM *vs.* 16.0% of non-diabetic patients; *P*=0.005), as shown in Table 2.

**Table 2:** Absolute and relative frequencies of the use of medications in the preoperative period according to the study group.

Variables	Original Cohort (n=2,688)		
	Diabetes		*P* value
	No (n=1,698)	Yes (n=990)	
Anticoagulant	8 (0.5)	6 (0.6)	0.639^[1]^
Acetylsalicylic acid	1,158 (68.2)	710 (71.2)	0.056^[1]^
Beta blocker	1,107 (65.2)	611 (61.7)	0.070^[1]^
Oral nitrates	1,106 (65.1)	577 (58.3)	<0.00^[1]^
Calcium-channel blocker	308 (18.1)	226 (22.8)	0.003^[1]^
Diuretics	431 (25.4)	282 (28.5)	0.079^[1]^
Angiotensin-converting enzyme inhibitors	790 (46.5)	500 (50.5)	0.046^[1]^
Angiotensin II receptor blockers	271 (16.0)	200 (20.2)	0.005^[1]^
Inotropic agents	3 (0.2)	2 (0.2)	1.000^[2]^
Statins	1,164 (68.6)	646 (65.3)	0.079^[1]^

[1] Descriptive level of probability determined by the chi-square test.

[2] Descriptive level of probability determined by the Fisher1s exact test.

Intraoperatively, diabetic patients underwent more procedures with the support of extracorporeal circulation (88.9% of patients with DM *vs.* 85.6% of non-diabetic patients; *P*=0.016), and a higher percentage of deaths occurred during surgery in patients with DM (0.5%) than in non-diabetic patients (0%; *P*=0.007) as can be observed in Table 3.

**Table 3:** Descriptive values [average ± standard deviation or n (%)] of surgical variables according to the study group.

Variables	Original Cohort (n=2,688)		
	Diabetes		*P* value
	No (n=1,698)	Yes (n=990)	
Emergency surgery/emergency	16 (0.9)	10 (1.0)	0.862^[3]^
Extracorporeal circulation	1,454 (85.6)	880 (88.9)	0.016^[2]^
Clamping time	43.5±19.1	44.3±19.8	0.332^[1]^
Intraoperative death	----	5 (0.5)	0.007^[3]^

[1] Descriptive level of probability by the Student1s t-test.

[2] Descriptive level of probability by the chi-square test.

[3] Descriptive level of probability by the Fisher1s exact test.

In the postoperative phase, patients with DM required greater use of transfused blood products (66.6% *vs.* 60.4% of non-diabetic patients; *P*=0.001) and had higher reintubation rates (IOT-Re) (6.5% of patients with DM *vs.* 3.4% of non-diabetic patients; *P*<0.001) (Table 4). Blood glucose was monitored during the first 24 hours after surgery. Patients with DM had higher average levels of blood glucose (197.2±36.6 *vs.* 166.9±26.1 mg/ dL for non-diabetic patients; *P*<0.001) and used intravenous insulin at higher rates (30.6% *vs.* 16.0% for non-diabetic patients; *P*<0.001).

**Table 4:** Descriptive values [average ± standard deviation or n (%)] of postoperative variables according to the study group. Original Cohort (n=2,688)

Variables	Original Cohort (n=2,688)		
	Diabetes		*P* value
	No (n=1,698)	Yes (n=990)	
Transfusion	1,025 (60.4)	659 (66.6)	0.001^[2]^
Orotracheal reintubation during hospitalization	58 (3.4)	64 (6.5)	<0.001^[2]^
Average blood glucose (24 hours)	166.9±26.1	197.2±36.6	<0.001^[1]^
Higher value of blood glucose (24 hours)	216.1±46.1	274.2±60.6	<0.001^[1]^
Lower value of blood glucose (24 hours)	126.5±26.2	131.5±37.7	<0.001^[1]^
Use of intravenous insulin (24 hours)	272 (16.0)	303 (30.6)	<0.001^[2]^
Readmission to the intensive care unit	117 (6.9)	70 (7.1)	0.856^[2]^
Time spent in intensive care unit	2.1±3.5	2.3±4.4	0.285^[4]^
Time of hospitalization	11.0±11.7	12.9±18.3	0.331^[4]^

[1] Descriptive level of probability of Student's t-test.

[2] Descriptive level of probability of chi-square test.

[4] Descriptive level of probability of Mann-Whitney U-test.

In terms of complications, the DM patient group showed higher rates of CKD (5.6% *vs.* 2.7% for non-diabetic patients; *P*<0.001), greater dialysis rates (2.2% *vs.* 0.7% for non-diabetic patients; *P*<0.001) and more infections (11.4% *vs.* 7.2% for non-diabetic patients; *P*<0.001), with mediastinitis (2.7% *vs.* 1.5% for non-diabetic patients; *P*<0.031) and urinary tract infections (3.2% *vs.* 1.2% for non-diabetic patients; *P*<0.001) being the most common (Table 5). Pneumonia was the most common postoperative infection, though it was not grouped in this study with the other infections: 7.7% of patients with DM *vs.* 4.0% of non-diabetic patients (*P*<0.001). Patients with DM also showed higher rates of mortality after one year, with a death rate of 9.1% compared with 5.6% in the non-diabetic patient group (*P*<0.001).

**Table 5:** Absolute and relative frequencies of complications according to the study group.

Complications	Original cohort (n=2688)		
	Diabetes		*P* value
	No (n=1,698)	Yes (n=990)	
Operative	60 (3.5)	35 (3.5)	0.998^[1]^
Perioperative acute myocardial infarction	23 (1.4)	9 (0.9)	0.304^[1]^
Cerebrovascular accident	21 (1.2)	21 (2.1)	0.075^[1]^
Acute renal failure	46 (2.7)	55 (5.6)	<0.001^[1]^
Dialysis	11 (0.7)	22 (2.2)	<0.001^[1]^
Infections	123 (7.2)	113 (11.4)	<0.001^[1]^
Mediastinitis	26 (1.5)	27 (2.7)	0.031 ^[1]^
Urinary tract infection	20 (1.2)	32 (3.2)	<0.001^[1]^
Prolonged mechanical ventilation	32 (1.9)	20 (2.0)	0.806^[1]^
Pneumonia	67 (4.0)	76 (7.7)	<0.001^[1]^
Arrhythmia	292 (17.2)	171 (17.3)	0.960^[1]^
Death within 30 days	49 (2.9)	40 (4.1)	0.105^[1]^
Death within 1 year	95 (5.6)	90 (9.1)	<0.001^[1]^

[1] Descriptive level of probability by the chi-square test.

To elucidate the role of DM in causing the complications found in this study, we performed a propensity score analysis, whose results are shown in Table 6. In this analysis, a total of 430 patients were analyzed (215 with DM and 215 without DM) with an average age of 61 years, of which 21.16% (91 of 430) were female. DM was not found to be a separate, independent risk factor for poor prognosis among these patients. In relation to postoperative complications, renal failure (occurring in 1.4% of DM patients *vs.* 0.9% of non-diabetic patients; *P*=1.0) was not found to be a significant factor after pairing. Infectious complications developed in 7.4% of patients with DM compared with 6.5% of non-diabetic patients (*P*=0.705). Finally, the mortality rates in patients with DM were evaluated after 30 days and found to be 1.4% diabetics *vs.* 2.3% for non-diabetic patients (*P*=0.724); after one year, mortality rates in patients with DM reached 3.3% *vs.* 5.6% in non-diabetic patients (*P*=0.245).

**Table 6:** Descriptive values [mean ± standard deviation or n (%)] of the variables after pairing according to the study group.

	Paired Cohort (n=430)		
	Diabetes		
Variables	No (n=215)	Yes (n=215)	*P* value
Age	61±10	62±9	0.700^[1]^
Male sex	169 (78.6)	170 (79.1)	0.906^[2]^
Body mass index (kg/m^2^)			
< 25	69 (32.1)	69 (32.1)	0.959^[2]^
25-30	117 (54.4)	115 (53.5)	
>30	29 (13.5)	31 (14.4)	
Dyslipidemia	94 (43.7)	115 (53.5)	0.043^[2]^
Chronic kidney disease	1 (0.5)	2 (0.9)	1.000^[3]^
Hypertension	207 (96.3)	207 (96.3)	1.000^[2]^
Previous stroke	4 (1.9)	2 (0.9)	0.685^[3]^
Peripheral arterial disease	7 (3.3)	16 (7.4)	0.054^[2]^
Cerebrovascular disease	4 (1.9)	4 (1.9)	1.000^[3]^
Prior coronary artery bypass surgery	2 (0.9)	1 (0.5)	1.000^[3]^
Previous acute myocardial infarction	94 (43.7)	104 (48.4)	0.333^[2]^
Arrhythmia	11 (5.1)	7 (3.3)	0.336^[2]^
Ejection fraction	67.2±9.7	65.1±12.5	0.230^[1]^
EuroSCORE	2.1±1.7	1.9±1.4	0.811^[4]^
Average blood glucose (24 hours)	170.7±27.2	192.0±32.1	<0.001^[1]^
Higher value of blood glucose	219.5±49.4	263.1±52.8	<0.001^[1]^
Use of intravenous insulin (24 hours)	39 (18.1)	63 (29.3)	0.007^[2]^
Time spent in the intensive care unit	2.1±4.4	1.6±0.9	0.297^[4]^
Time of hospitalization	11.6±19.7	9.8±10.6	0.088^[4]^
Surgeries	5 (2.3)	2 (0.9)	0.449^[3]^
Perioperative acute myocardial infarction	3 (1.4)	_	0.248^[3]^
Cerebrovascular accident	1 (0.5)	3 (1.4)	0.623^[3]^
Acute renal failure	2 (0.9)	3 (1.4)	1.000^[3]^
Dialysis	1 (0.5)	_	1.000^[3]^
Infections	14 (6.5)	16 (7.4)	0.705^[2]^
Mediastinitis	4 (1.9)	3 (1.4)	1.000^[3]^
Urinary tract infection	3 (1.4)	6 (2.8)	0.503^[3]^
Prolonged mechanical ventilation	2 (0.9)	_	0.499^[3]^
Pneumonia	6 (2.8)	6 (2.8)	1.000^[2]^
Arrhythmia	32 (14.9)	31 (14.4)	0.892^[2]^
Death within 30 days	5 (2.3)	3 (1.4)	0.724^[3]^
Death within 1 year	12 (5.6)	7 (3.3)	0.245^[2]^

[1] Descriptive level of probability by the Student's t-test.

[2] Descriptive level of probability by the chi-square test.

[3] Descriptive level of probability by the Fisher's exact test.

[4] Descriptive level of probability by the nonparametric Mann-Whitney U-test.

## DISCUSSION

In this study, patients with DM had higher rates of mortality and morbidity (mainly renal disease and infectious) after CABG. However, after performing propensity scoring, DM was not found to be an independent predictive factor for these findings.

Patients with DM who underwent CABG more often suffered from acute renal failure (5.6%). These data were confirmed by Adalsteinsson et al.^[14]^ performed in their retrospective study, which enrolled 1,626 patients who underwent CABG surgery in Iceland from 2001 to 2012. They compared 261 (16%) patients with DM with 1,365 non-diabetic patients, and 14% *vs.* 9% of these patients, respectively, developed acute renal failure (*P*=0.02). In other analyses, Kubal et al.^[15]^ showed that insulin-dependent DM was associated with an increased incidence of acute renal failure (adjusted odds ratio, 4.15; *P*=0.002).

The predisposition of patients with DM to infectious complications after cardiac surgery has frequently been suggested by different authors^[16-21]^ but with some exceptions^[6,14,17,22]^, in which no trend of incidence was detectable. The main reasons for the predisposition to the cited infections are due to the strong association between DM and angiopathy, neuropathy and hyperglycemia. Diabetic patients present an increased risk for postoperative infections due to depreciated host defense mechanisms, such as impaired as wound healing and granulocyte function, decreased cellular immunity, impaired complement function, and reduced immune response, which may be influenced by the glycemic control^[20-24]^.

In addition to increasing the risk for postoperative infections in CABG, DM was associated with a significantly higher incidence of late mortality (up to one year) and related cardiac events. In our study, intraoperative mortality was also higher in patients with DM compared with non-diabetic patients (0.5% *vs.* 0%; *P*=0.007). Recent studies by Szabo et al.^[25]^ e Kubal et al.^[15]^ concur with our findings, reporting an intrahospital mortality of 2.6% and 2.4% for their DM patient populations, respectively. However, Kubal et al.^[15]^, after adjusting the propensity score, showed that there was no longer a significant association between DM and hospital mortality, as in our study. Therefore, the 30-day mortality rate in our study was 4.1% in patients with DM *vs.* 2.9% in non-diabetic patients (*P*=0.105). Similar findings were also obtained by Szabo et al.^[25]^, who reported a mortality rate 2.6% for DM patients *vs.* 1.6% for non-diabetic patients (*P*=0.15).

Our study did not assess subtypes of DM patients, because DM type I (DM1) may have a higher severity, as demonstrated in another study^[26]^, in which patients with DM1 presented a two-fold increase in the risk of death compared to that in non-diabetic patients, and DM type 2 patients had a small but significant increased risk of death than that of the same population. Another possible explanation for the null association between DM and mortality may be the low number of patients used in the comparison.

The mortality rate did not remain an independent risk factor after the propensity score analysis. In our analysis, patients with DM presented more comorbidities, such as more-severe atherosclerotic disease and more complications. Therefore, they had a more urgent indication for surgery and a significantly higher usage of extracorporeal circulation, which can contribute to a greater chance of complications after surgery.

This study had several limitations. First, this was an observational study, and we can speak of causality only in terms of association. At the time of data collection, this analysis had not been planned, and for this reason, this study was not able to evaluate the outcomes for specific types of DM. The fact that the data were collected from medical records may have affected the quality and completeness of the data, which depended on notes from health professionals. In addition, this study used a database from a single institution (one center), and although compiled by different surgical teams, this method limits the generalizability of our results.

## CONCLUSION

Finally, we found that patients with diabetes were at higher risk for postoperative complications and mortality after undergoing CABG, however, in our study, diabetes did not explain the poor prognosis of these patients after pairing this factor with the propensity score.

**Table:** 

**Authors' roles & responsibilities**	
KAQS	Conception and design study; realization of operations and/or trials; statistical analysis; analysis and/or data interpretation; manuscript writing or critical review of its content; final manuscript approval
BB	Conception and design study; realization of operations and/or trials; statistical analysis; analysis and/or data interpretation; manuscript writing or critical review of its content; final manuscript approval
AGS	Manuscript writing or critical review of its content; final manuscript approval
FAAC	Manuscript writing or critical review of its content; final manuscript approval
